# Effects of dupilumab in type 1 neurofibromatosis coexisting with severe atopic dermatitis^☆^^☆☆^

**DOI:** 10.1016/j.abd.2020.10.006

**Published:** 2021-07-16

**Authors:** Camilla Chello, Alvise Sernicola, Giovanni Paolino, Teresa Grieco

**Affiliations:** Dermatology Unit, Sapienza University of Rome, Rome, Italy

## Abstract

Neurofibromatosis type 1 still lacks established treatment options aimed at controlling the progression of neurofibromas as well as effective therapy for the neurogenic itch associated with the disease. We report the case of a 30-year-old Caucasian woman with type 1 neurofibromatosis coexisting with severe refractory atopic dermatitis. Dupilumab, a novel anti-IL-4 receptor alpha monoclonal antibody, the first biologic agent approved for atopic dermatitis, was the drug of choice in this case. We observed remission of atopic dermatitis and a remarkable reduction in the size and swelling of neurofibromas and in the related pruritus, that became evident after one month of treatment. After 18 months of therapy, no new neurofibromas were detected and preexistent lesions showed no increase in size. These findings are consistent with the hypothesis that dupilumab, a potent anti-inflammatory drug, may have a positive effect on type 1 neurofibromatosis by stopping the progression of preexisting neurofibromas and the onset of new lesions.

Dear Editor,

We report the case of a 30-year-old Caucasian woman with type 1 neurofibromatosis (NF1), who came to medical attention for the recent worsening of a concomitant severe form of atopic dermatitis (AD). The patient presented typical features of NF1: axillary and inguinal freckles, café-au-lait spots, multiple subcutaneous neurofibromas, Lisch nodules, spinal alterations with scoliosis. AD was characterized by a generalized pattern and predominant involvement of the face with eyelid eczema and ectropion (Fig. 1). Eczema area severity index (EASI) score was 30 and dermatology life quality index (DLQI) was 25, corresponding to a severe form of the disease. Due to the inefficacy of previous treatments with systemic steroids and cyclosporine in achieving clinical improvement of atopic dermatitis, the patient started therapy with dupilumab at a standard approved dosage of 600 mg subcutaneously followed by 300 mg every two weeks, according to current guidelines. Four weeks after the initiation of therapy, we observed improvement in the signs and symptoms of AD (EASI 4) (Fig. 2). As a collateral finding, we also observed a reduction in the size and swelling of the neurofibromas (Fig. 3). After 16 weeks, we assessed complete remission of AD and no progression of NF1, in terms of number and size of neurofibromas, with an overall improvement in the quality of life of the patient (DLQI 0). After 18 months of treatment, the cutaneous burden of NF1 remained stable.

**Figure 1:**
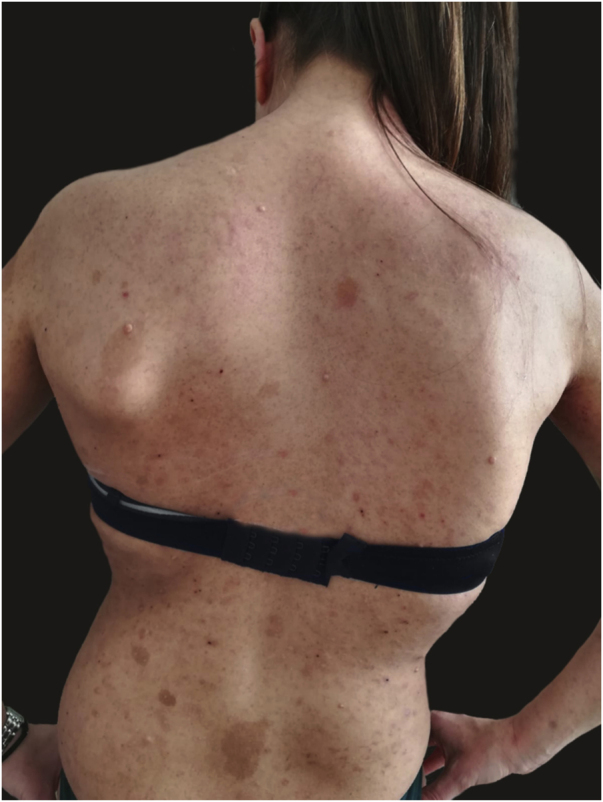
Clinical signs of type 1 neurofibromatosis and atopic dermatitis. Eczematous lesions, with erythema, oozing and crusting, several cafe au lait spots, freckles, cutaneous neurofibromas.

**Figure 2:**
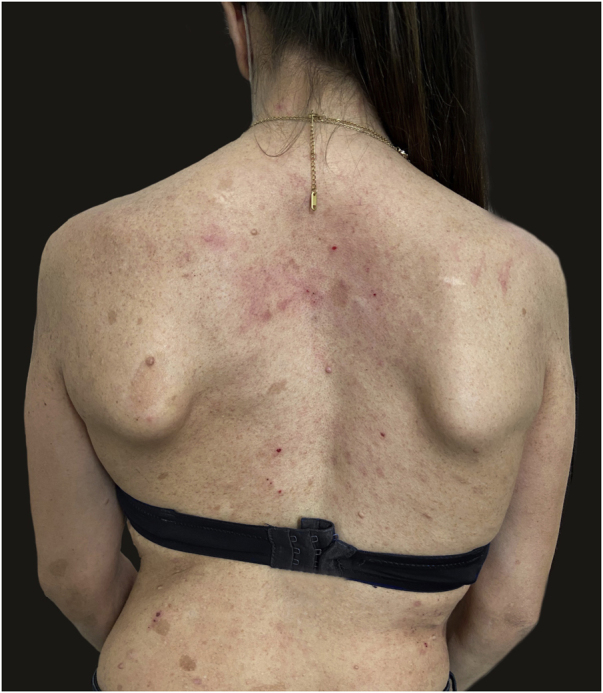
Remission of atopic dermatitis after 4 weeks of treatment with dupilumab. Residual eczematous lesions, as seen on the neck, determined an EASI score of 4.

**Figure 3:**
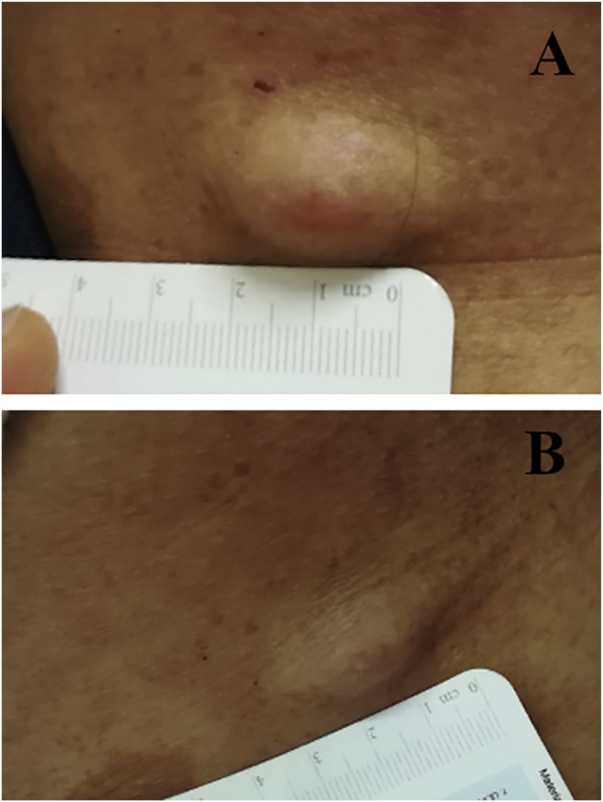
(A), Cutaneous neurofibroma before starting treatment with dupilumab. (B), The same lesion shows evident reduction in swelling and firmness after four weeks of therapy.

In our patient, dupilumab proved to be effective both in the management of severe AD and in neurofibromas, achieving stabilization of the disease at one year. The possible effectiveness of the drug on NF1 may reside in the molecular pathology of neurofibromatosis. Fibroblasts and mast cells are key players in the promotion of tumor growth in the neurofibroma microenvironment, as well as in wound healing and scar formation.^1^, ^2^ As previously reported, the activation of IL-4 and IL-13 pathways in fibroblasts, mediated by JAK/STAT intracellular signaling, leads to excessive collagen production, which is responsible for neurofibroma development.^3^ In regard to NF1, we hypothesize that anti-IL-4 receptor monoclonal antibody dupilumab may inhibit the growth of neurofibromas, interfering with IL-4 and IL-13 binding to type I and type II receptors expressed on mast cells and fibroblasts. This is consistent with the mechanism of action previously described in AD.^4^ To date, pharmacological treatments for neurofibromas in NF1 are still lacking. Moreover, there is no previous reported evidence of the effect of dupilumab in the treatment of NF1. This is probably also due to the paucity of studies highlighting the association between the two disorders. Indeed, only one study reported the co-existence of concomitant AD in 18% of 227 NF1 patients, but these data are not confirmed by further evidence in the current literature.^5^

Our experience could be helpful in the management of NF1, underlining the beneficial anti-inflammatory effect of this biological drug on the neurocutaneous disease, but we are conscious that pathogenetic studies of cytokine interactions and immune signaling pathways as well as RCTs are needed in order to investigate the use of dupilumab in NF1 treatment.

## Financial support

None declared.

## Authors’ contributions

Camilla Chello: Study conception and planning; preparation and writing of the manuscript.

Alvise Sernicola: Study conception and planning; preparation and writing of the manuscript.

Giovanni Paolino: Manuscript critical review; preparation and writing of the manuscript.

Teresa Grieco: Study conception and planning; manuscript critical review.

## Conflicts of interest

None declared.
